# COVID-19-Associated Tension Pneumothorax in Mechanically Ventilated Patients: A Case Series

**DOI:** 10.7759/cureus.26216

**Published:** 2022-06-22

**Authors:** Muhammad Ali Raza, Michael W Figart, Krithika Suresh, Talha Mehmood

**Affiliations:** 1 Internal Medicine, Conemaugh Memorial Medical Center, Johnstown, USA

**Keywords:** severe acute respiratory syndrome coronavirus 2 (sars-cov-2), tension pneumothorax in covid-19, covid-associated pneumothorax, tension pneumothorax on pocus, tension pneumothorax

## Abstract

Severe acute respiratory syndrome management secondary to coronavirus (SARS-CoV-2) has been overwhelming for healthcare systems. Patients with SARS-CoV-2 infection can present with symptoms ranging from a mild flu-like illness to acute respiratory distress syndrome (ARDS). Patients who develop coronavirus disease 2019 (COVID-19) infection and present with hypoxic respiratory failure requiring mechanical ventilation typically follow ARDS physiology. Many of them develop complications including pneumothorax, pneumomediastinum, and pneumopericardium. In this case series, we present multiple instances where patients with severe COVID-19 infections developed tension pneumothoraces during their hospital course.

## Introduction

Patients who develop coronavirus disease 2019 (COVID-19) infection and present with hypoxic respiratory failure requiring mechanical ventilation typically follow acute respiratory distress syndrome (ARDS) physiology [1]. In patients with COVID-19, tension pneumothorax is a potential cause of abrupt decompensation. It is critical to recognize tension pneumothoraces as early as possible, due to high mortality rate of up to 91%, if not diagnosed and treated promptly [2].

## Case presentation

Case 1

**Figure 1:**
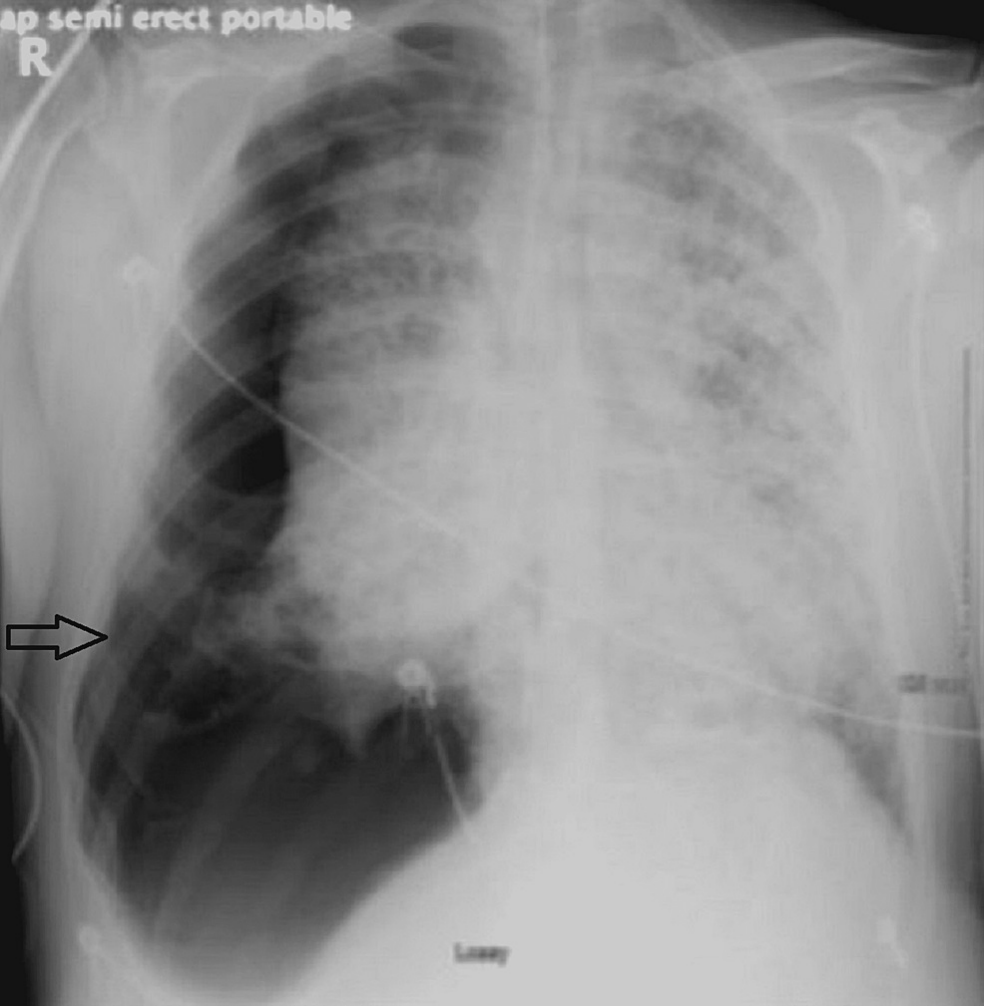
Right-sided tension pneumothorax

A 63-year-old male with a medical history significant for asthma, hyperlipidemia, prostate cancer, and cluster headaches presented to the emergency department (ED) with worsening shortness of breath (SOB). The patient tested positive for SARS-CoV-2 via polymerase chain reaction (PCR) six days prior to presentation. Because of being hypoxic on room air, the patient was supplemented with oxygen via nasal cannula. A computed tomography (CT) scan of the chest with contrast revealed widespread pneumonitis with mediastinal adenopathy. After being admitted to the medical floor, he was given a 10-day course of dexamethasone and a five-day course of remdesivir. Despite these measures, his respiratory condition worsened, and he required non-invasive positive pressure ventilation (NIPPV). He continued to deteriorate on NIPPV and was transferred to the intensive care unit (ICU) for escalation of care on day 14 of his hospital stay. In the ICU, he was given tocilizumab and was diagnosed with severe ARDS, and prone position mechanical ventilation was initiated. Despite maximum oxygen of 100%, he remained hypoxic and continued to deteriorate despite increasing his positive end-expiratory pressure (PEEP) to 14 cm H_2_O. While in the prone position, his saturations plummeted to the 50s, due to which a chest x-ray was obtained and it revealed a right-sided tension pneumothorax (Figure 1).

Needle decompression was performed immediately. The patient went into cardiac arrest with pulseless electrical activity. Cardiopulmonary resuscitation (CPR) was initiated and a surgical chest tube was placed. The patient's relatives requested that resuscitative efforts be held, and the patient’s care was eventually transitioned to focus on his comfort only.

Case 2

**Figure 2:**
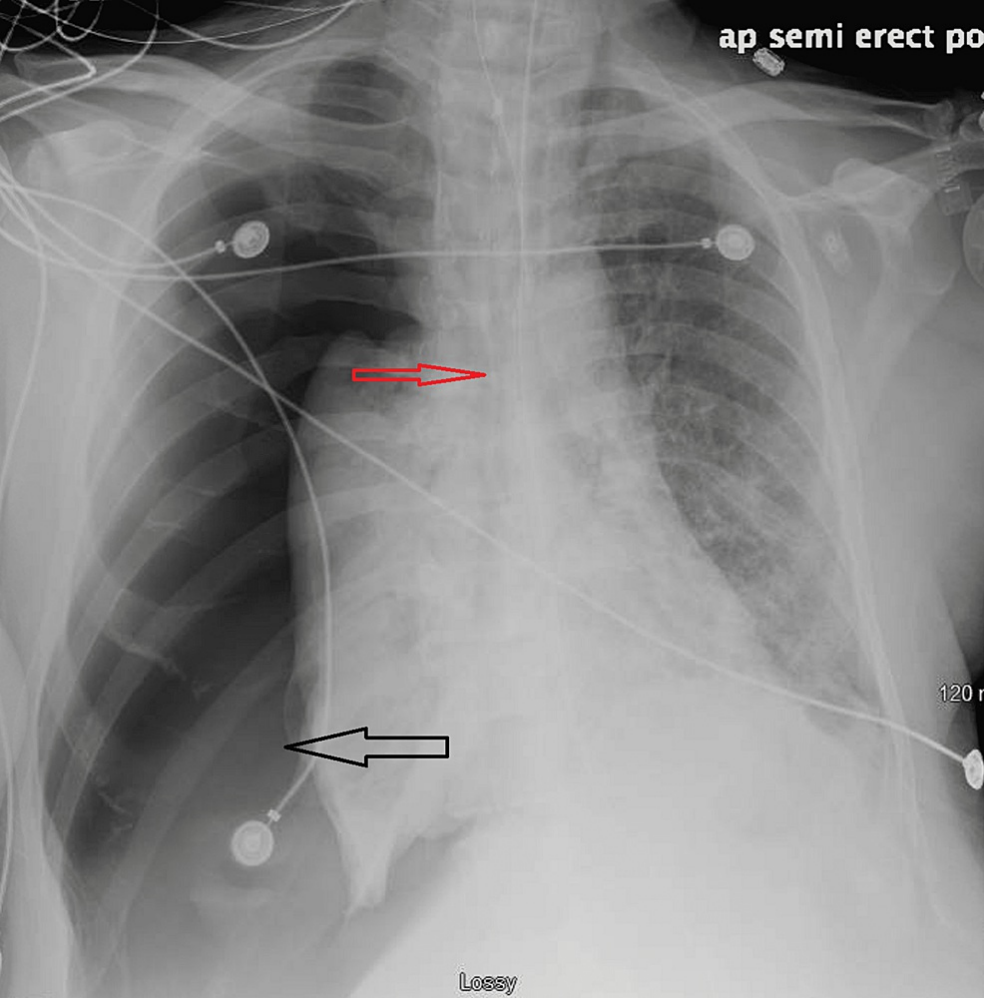
Significant right-sided tension pneumothorax (black arrow) with displacement of mediastinal structures towards the opposite side of pneumothorax (red arrow)

A 61-year-old male with a past medical history of diabetes mellitus, gout, and nicotine dependence presented to our facility with worsening respiratory status. The patient tested positive for SARS-CoV-2 via PCR eight days prior and presented to an outside hospital five days prior to transfer. He was treated for acute hypoxic respiratory failure with superimposed bacterial pneumonia with dexamethasone, azithromycin, and ceftriaxone. On the eighth day of his hospital stay, his respiratory status deteriorated, and he was intubated and transported to our facility. He had significant hemodynamic instability requiring vasopressor support and furosemide for profound volume overload. Due to deteriorating hypoxic respiratory failure, his antibiotics were escalated to piperacillin-tazobactam and vancomycin. On day 25, the patient's vasopressor requirements increased dramatically and oxygen saturations deteriorated, necessitating higher ventilator settings. A bedside ultrasound revealed pneumothorax, which was confirmed with a chest x-ray (Figure 2).

Needle decompression was done, followed by chest tube insertion which improved hemodynamic status. Unfortunately, given his profound co-morbidities, including worsening renal function, and concern for bleeding goals of care discussions were held, and his family requested that comfort measures be pursued.

Case 3

A 72-year-old male with a history of coronary artery disease, hypertension, and diabetes mellitus presented to the ED with SOB after four days of testing positive for SARS-CoV-2 via PCR. He was treated with azithromycin and dexamethasone by his primary care provider at home, but his symptoms did not improve. On arrival, his oxygen saturation was in the 70s while breathing ambient air. He was placed on a nonrebreather mask and admitted to the medical floor. Remdesivir and dexamethasone were initiated. Due to his worsening respiratory status, he was placed on a high-flow nasal cannula which led to minimal improvement. On day five of his hospital stay, NIPPV was initiated with a PEEP of 10. He was transferred to the ICU for further treatment where he was empirically covered for possible superimposed bacterial infection with vancomycin and piperacillin-tazobactam. Due to new-onset atrial fibrillation and a high d-dimer, full-dose anticoagulation with heparin infusion, and aggressive diuresis were also initiated. He was eventually intubated due to persistently low oxygen saturation despite four days of NIPPV support. Owing to rapidly worsening acute kidney injury, he was placed on continuous renal replacement therapy. Moreover, his septic shock necessitated vasopressor administration. He eventually developed severe ARDS and was placed in prone position while being mechanically ventilated. His norepinephrine requirement increased dramatically from 0.5 mcg/kg/min to 3 mcg/kg/min on day 17 of his hospital stay. Vasopressin was added. His oxygen saturation dropped into the 50s, so he was taken off the ventilator and manually bagged, but he did not improve. A bedside ultrasound revealed tension pneumothorax. 

Needle decompression was performed and a surgical chest tube was inserted. His vital signs improved, and he was weaned off the pressors. He did develop a non-ST-elevation myocardial infarction (NSTEMI), but due to his poor health, he was not a candidate for cardiac catheterization. The patient’s family ultimately decided to pursue comfort measures.

## Discussion

Patients who develop severe coronavirus disease 2019 (COVID-19) infection and present with hypoxic respiratory failure requiring mechanical ventilation typically follow ARDS physiology [1]. In patients with COVID-19, tension pneumothorax is a potential cause of abrupt decompensation. It is critical to recognize tension pneumothoraces as soon as possible because of the high mortality rate of up to 91% if not diagnosed and treated promptly [2]. Tension pneumothorax involves air accumulation between the parietal and visceral pleura because of a one-way air admission with no outlet. The disorder causes the ipsilateral lung to collapse and the surrounding structures, including vasculature, to shift away from the affected pleura. This can result in a considerable drop in preload, resulting in obstructive shock with severe hemodynamic instability [3].

To diagnose tension pneumothorax, a physical examination and point-of-care ultrasonography (POCUS) are required. The sensitivity of chest x-ray ranges from 50% to 80%, and it can be improved by taking upright x-rays [4]. Ultrasound, on the other hand, can be completed in less time, allowing for faster treatment. The POCUS examination requires the patient to be supine and the probe to be placed between the third and fourth ribs. The visceral and parietal pleural layers normally slide back and forth (Video 1). The absence of this sign most likely signifies the presence of pneumothorax [4].

**Video 1:** Normal lung sliding present on POCUS POCUS: point-of-care ultrasonography

In a study of ED physicians comparing POCUS and chest x-rays to diagnose pneumothorax, it was discovered that POCUS recognized 25 of 29 pneumothoraces, whereas the average chest x-ray detected just eight [5]. The gold standard for diagnosing a pneumothorax is still a CT scan; however, the acuity of the situation makes it rarely feasible. Tension pneumothorax must be treated swiftly to avoid further deterioration of the patient's clinical status in a step-wise manner. First and foremost, the circulation, airway, and breathing need to be stabilized. If the patient is clinically unstable, needle decompression should be undertaken to allow for quick lung re-expansion and an improved clinical state [6].

A notable case series of COVID-19 patients was presented by Chen et al., in which 17% experienced acute respiratory distress syndrome (ARDS) and 1% developed pneumothorax [7]. Brogna et al. identified loculated pneumothorax as a consequence of COVID-19 infection, which is commonly associated with ARDS in mechanically ventilated patients [8]. Pneumothorax was found to be a rare complication of COVID-19 infection in a large retrospective study by Zantah et al., affecting six of the patients in their study, and mechanical ventilation was noted to be a key risk factor for the development of pneumothorax, as four of the six patients had received mechanical ventilation [9]. Pneumothorax was reported as a complication of COVID-19 by Martinelli et al., with the finding that the incidence was higher in males, despite the fact that it did not appear to be an independent marker of poor prognosis [10]. Guo et al., on the other hand, published a retrospective investigation of 21 COVID-19 patients who had spontaneous pneumothoraces, noting that it may be related to a worse prognosis because of high mortality (42.9%) in their case series [11].

Our case series revealed that COVID-19 infection may be associated with a higher prevalence of pneumothorax. Of note, all of our patients were male. It was observed that those who developed tension pneumothorax had a worse prognosis and clinical outcomes due to hemodynamic deterioration despite timely recognition and treatment.

## Conclusions

According to our study of case series, tension pneumothorax is a probable consequence of severe COVID-19 infection. If a patient with COVID-19 requiring mechanical ventilation suddenly deteriorates, one must be vigilant in recognizing this catastrophic consequence, as early detection and treatment could be lifesaving. We further stress the importance of POCUS in timely diagnosis of pneumothorax. Because all the patients in this case series had received mechanical ventilation, it appears to be a risk factor for the development of pneumothorax. However, further research is needed to elucidate the pathogenesis of pneumothorax in patients with severe COVID-19.
